# Limitations in the comparison of the Brazilian National Dietary Surveys of 2008–2009 and 2017–2018

**DOI:** 10.11606/s1518-8787.2021055003365

**Published:** 2021-11-22

**Authors:** Renata Muniz Rodrigues, Eduardo De Carli, Marina Campos Araújo, Eliseu Verly, Dirce Maria Lobo Marchioni, Ilana Nogueira Bezerra, Amanda de Moura Souza, Edna Massae Yokoo, Rosangela Alves Pereira, Rosely Sichieri

**Affiliations:** I Universidade do Estado do Rio de Janeiro Instituto de Medicina Social Rio de Janeiro RJ Brasil Universidade do Estado do Rio de Janeiro. Instituto de Medicina Social. Rio de Janeiro, RJ, Brasil; II Universidade de São Paulo Faculdade de Saúde Pública Departamento de Nutrição São Paulo SP Brasil Universidade de São Paulo. Faculdade de Saúde Pública. Departamento de Nutrição. São Paulo, SP, Brasil; III Fundação Oswaldo Cruz Escola Nacional de Saúde Pública Sergio Arouca Departamento de Epidemiologia e Métodos Quantitativos em Saúde Rio de Janeiro RJ Brasil Fundação Oswaldo Cruz. Escola Nacional de Saúde Pública Sergio Arouca. Departamento de Epidemiologia e Métodos Quantitativos em Saúde. Rio de Janeiro, RJ, Brasil; IV Universidade Estadual do Ceará Centro de Ciências da Saúde Fortaleza CE Brasil Universidade Estadual do Ceará. Centro de Ciências da Saúde. Fortaleza, CE, Brasil.; V Universidade Federal do Rio de Janeiro Instituto de Estudos em Saúde Coletiva Rio de Janeiro RJ Brasil Universidade Federal do Rio de Janeiro. Instituto de Estudos em Saúde Coletiva. Rio de Janeiro, RJ, Brasil; VI Universidade Federal Fluminense Departamento de Epidemiologia e Bioestatística Niterói RJ Brasil Universidade Federal Fluminense. Departamento de Epidemiologia e Bioestatística. Niterói, RJ, Brasil; VII Universidade Federal do Rio de Janeiro Departamento de Nutrição Social e Aplicada Rio de Janeiro RJ Brasil Universidade Federal do Rio de Janeiro. Departamento de Nutrição Social e Aplicada. Rio de Janeiro, RJ, Brasil

**Keywords:** Dietary surveys, methods, Data Collection, Food Composition Table, Use of Procedures and Techniques

## Abstract

**OBJETIVE:**

To present particular characteristics of two Brazilian National Dietary Surveys (*Inquéritos Nacionais de Alimentação* - INA) and the methodology used to better compare their data.

**METHODS:**

This study details the differences between both INA conducted by the Brazilian Institute of Geography and Statistics (IBGE) in 2008–2009 and 2017–2018. We present the alterations in data collecting methods and food composition tables as well as the analysis strategies recommended to obtain such data. A validation study with 95 participants of the third wave of the Longitudinal Study of Adult Health assessed the measurement error associated with the procedures adopted in the 24-hours dietary recall of INA 2017–2018. The reference standards were urinary protein recovery, sodium, and potassium biomarkers. Different strategies were used in the analysis of INA to compare two essential dietary items that had their collection method changed: fats and sugars.

**RESULTS:**

The validation study indicated lower underreport in the most recent survey with higher means of energy intake. The correlation of means for the 24-hours recalls with their respective biomarkers was 0.58 for proteins, 0.31 for potassium, and 0.30 for sodium. Comparing the food composition tables used in both surveys with the data obtained by INA 2008-2009, the mean variation of energy, macronutrients, and minerals was lower than 15%, except for trans fats and selenium, which had means 40% and 52% lower in the *Tabela Brasileira de Composição de Alimentos* (TBCA - Brazilian Food Composition Table). INA 2017–2018 presents lower means for added sugar, using a generic question about the frequency of sugar consumption as a measure for sugar as an additional item.

**CONCLUSION:**

The methodological changes promoted in the most recent INA enhanced food groups and nutrients intake estimation, adding detailed and specific data in dietary habits reports.

## INTRODUCTION

Until 2008, estimations of food consumption and its periodic changes in Brazil relied on food availability per household data obtained by the *Pesquisas de Orçamentos Familiares* (POF – Family Budget Researches), which were conducted periodically since the 1970s by the Brazilian Institute of Geography and Statistics (IBGE). These estimations depend on aggregate data, such as census or sample strata; therefore, they are indirect data. Besides, household food availability data do not consider away-from-home food intake, which leads to consumption underestimation^1^. Although aggregate estimations allow national and international comparisons, Brazil only collected individual consumption data after the first decade of the 21st century, which had national representativeness enabling the evaluation of characteristics such as consumption per age range, gender, and other individual factors.

The POFs of 2008–2009 and 2017–2018 included a modulus called *Inquérito Nacional de Alimentação* (INA – National Dietary Survey), which obtained food consumption data for all individuals with at least 10 years old with a probabilistic subsample of the households selected for the POF’s original sample. The survey of 2008–2009 studied 13,569 houses, corresponding to 24.3% of the overall POF sample^2^. In 2017–2018, the proportion of households in the INA subsample was 34.7%, comprising 20,112 households^3^. Both surveys allow estimations according to the country regions (North, Northeast, Southeast, South, and Center-West) and urban or rural residence. Differently from household food availability data, the surveys estimated away-from –home food consumption and characterized the diet according to the intake of foods, food groups and eating patterns. They also evaluated energy and nutrient intakes, and micronutrient intake inadequacy. Considering the changes between both surveys in the data collection methods and the food centesimal composition tables, this study aims to present the characteristics of both INA and the methodology applied to compare their data, allowing the analysis of food consumption variation.

## METHODS

### Data-Collecting Instruments

In both surveys, food and beverage intake was evaluated in two nonconsecutive days, selected during the week in which the researcher visited the household. Each household’s inhabitants with 10 years old or more were evaluated to represent all days of the week.

The instrument used in 2008–2009 was the food record. Individuals were guided to report their food and beverage intake, the cooking method, the measurement units, the amount, time and place of their meals. All food and beverage consumed during the selected days were written down on appropriate forms. The notes were revised by the researcher and entered into a digital database still in the household. When the participant had any problem to fulfill the food record, a relative could be indicated to help. In 2017–2018, the 24-hours recall (24h-R) was used. The researchers conducted in-person interviews about all food and beverage (including water) consumed during theday beforethe interview. A software desingned for tablets specificallyfor the research was used to support the interviews administered following sequential stages to obtain information on food intake, based on an Automated Multiple-Pass Method^4^. While the interviewee reported uninterruptedly the data, the interviewer made a quick list on paper, including hours and occasions of consumption. Based on this quick list, the interviewer registered the data in the software, also detailing the data regarding food and beverage intake.

The option to chance the data collection method from food record to the 24h-R was, primarily, by the almost universal use of this instrument in population-based researches, since it is considered the method with the lower probability of systematic error^5^. Additionally, the validation study of the instrument used in 2008–2009, which used as a gold standard the doubly labeled water method^6^, indicated better performance of the 24h-R compared to the food record. The Box details the changes in the data-collecting methods between the two surveys. In both surveys, the database software alerted when the individual reported less than five items or when three or more hours had passed with no food item reported. In such cases, the researcher should confirm if all food consumed in the days studied were reported and verify possible omissions or incomplete data.

**Box:** Chart-summary of the data-collecting instruments of the Brazilian National Dietary Surveys, 2008–2009 and 2017–2018.

	INA 2008–2009	INA 2017–2018
**Data-collecting Instrument**	Individual dietary assessment	25 hours recall
**Food items**	1,503	1,832
**Place of consumption**	Within or outside the household	Inside the house or prepared at home. School.
		Kilo restaurant.
		Cafeteria, bar, snack bar, fast food. Restaurant – others.
		Street vendors.
		Outside the household – others.
**Types of preparation**	15 options	9 options
**Perceived units of measurement**	106 options (including g, kg, ml, and l)	64 options
**Occasion of consumption**	Not included	Breakfast; lunch; snack; dinner; supper; other occasion.
**Added items**	Not included	12 items: oil, butter/margarine, sugar, sugar sweetener, honey, molasses, mayonnaise, ketchup, mustard, soy sauce, grated cheese, and cream.

The food and beverage intake database had 1,503 items in 2008–2009 and 1,832 items in 2017–2018. The new list of food was updated based on data from the *Estudo de Riscos Cardiovasculares em Adolescentes* (Adolescents’ Cardiovascular Risk Study)^7^ and surveys performed to update the GloboDiet study^8,9^. In both surveys, the software demanded for each food or beverage intake the quantity consumed (measurement unit and amount). In 2017–2018, an extensive review and update of the table for measures retorted for foods consumed in Brazil in the POF 2008–2009^10^ was carried out to elaborate the database of quantities in grams or milliliters. In 2008–2009, the software allowed the reporting of variations in kilograms and liters of 106 measurement units. In 2017–2018, 64 measurement units were available. More details can be found in the reports of both surveys^3,10^. Both surveys had questions regarding the place and hour of the consumption, from 0 to 23 hours without decimal points. In 2008–2009, for the place of consumption, “At-home” was used when the food came from the household, and “Any-from-home” when food was obtained or consumed outside the household. In 2017–2018, the options available to report the place of consumption were: household, work, school, restaurant, cafeteria, street vendors, and other place outside the household.

For selected foods, particularly vegetables and meat, the cooking method was asked. In 2008–2009, the software presented 15 options to report the cooking method; in 2017–2018, 9 options. The reduction occurred because certain cooking methods, such as breaded, white or red sauce, with garlic and oil, porridge, and soup were incorporated to the corresponding food items. For food that do not present significant variations in their cooking method, such as bread, milk, and yogurt, the option “not applicable” was used in both surveys in the question ‘cooking method’.

The survey of 2017–2018 innovated by asking the interviewee to indicate the eating occasion and to report the addition of items. The following options were available to report the eating occasion: “breakfast”, “lunch”, “snack”, “dinner”, “supper”, and “other occasion”. In 2008–2009, items added were reported as food. However, in 2017–2018, a list of 12 items was available to report items that usually are added to foods, for instance, butter or margarine to bread, corn or biscuits and mustard to sandwiches, salad or potato. The options were specific for each food item. When appropriate, the response options for each item added were “yes”, “no”, or “nor applicable”. There was no limit in the number of items added to a food. The amount of each item added was not asked.

### Instruments Validation

As aforementioned, a validation study in 2008–2009 conducted with a convenience sample, analyzing doubly labeled water, found an underreport of energy intake of 30%^6^. For the 2017–2018 survey, a validation study was conducted using the recorvery biomarkers, specifically, urinary protein (nitrogen), sodium, and potassium, as reference to evaluate measurement errors associated with the 24h-R procedures.

The study investigated 95 individuals (54,2% women) between 44 and 72 years old which participated in the third wave of the Longitudinal Adult Health Study (ELSA-Brasil) in São Paulo. These participants reported body weight stability in the last six months and no records of disease or continuous use of medicament or vitamin-mineral supplements. Means of daily protein, sodium, and potassium intake, adjusted by intrapersonal variability, were measured based on two 24h-R and two 24 hours urine samples. 83 participants provided the biomarkers of urinary recovery.

### Food Composition Data

In 2008–2009, to estimate energy and nutrient intake, a food composition table of foods reported in INA^11^ was elaborated based on the compilation of renowked food composition tables such as the *Tabela Brasileira de Composição de Alimentos* (TACO) (NEPA, 2006) and the Nutrition Data System for Research (NDSR) of the University of Minnesota (NDS-R, 2003). In 2017–2018, the Brazilian Food Composition Table (Brazilian Food Composition Table - TBCA version 7.0) was used to estimate nutrient intake. The TBCA is provided by the *Rede Brasileira de Dados de Composição de Alimentos* (Brazilian Network of Food Composition Data), Universidade de São Paulo, and the *Centro de Pesquisa em Alimentos* (Center of Food Research), and is available on http://www.tbca.net.br/^12^.

The TBCA is adequate to the national survey context and has its quality and reliability guaranteed by the guidelines and criteria to generate, compile, and use food composition data established by the International Network of Food Data Systems (INFOODS), of the Food and Agriculture Organization of the United Nations (FAO)^12,13^.

Differences between the surveys of 2008–2009 and 2017–2018, such as alterations in the evaluation method and the use of a new food composition table, imply in technical challenges to compare two important items of food intake: fats and sugars. In the first INA, the nutritional composition of cooked meats did not included any type of fat addition in the cooking process^11^. Nevertheless, assuming that, usually, meats are cooked with kind of fat, programming routines were adopted to change the cooked meat to braised meat, aiming to approximate the nutritional composition of this type of meat when prepared with oil^2^. INA 2017–2018 reviewed the types of preparation, and the cooking method ‘cooked’ presented the options with or without fats. Thus, it was possible to identify individuals who used or not fats while cooking meat^3^.

The analysis of the evolution of sugar intake was technically more challenging. Both surveys included a generic and qualitative question about the sweetener most often used in food and beverage (sugar or a sweetener, both, or none). However, they did not identify which items were sweetened. In INA 2008–2009, such answers were considered in the category of energy and nutrient intake, considering a proportion of 10% of added sugar in beverages for individuals who reported using only sugar, and 5% for those who reported using sugar and sweetener^11^. However, in INA 2017–2018, sugar was included among the 12 options of additions possible for each food item. This question was also qualitative, and a standard percentage of 10% was established for added sugar in the total amount of food or beverage reported^3^.

## RESULTS

### Validation Study

For the 95 participants, the mean of energy intake for both recalls was 2,078 kcal with a standard deviation (SD) of 663. This value was 2,339 kcal for men (n = 43), SD = 691, and 1,863 kcal for women (n = 52), SD = 560.

The results for biomarkers using the same database of INA 2017–2018 indicate variation in the dietary measurement errors between nutrients and genders. For proteins, the 24h-R mean did not differ significantly from the obtained with the biomarker, although an underreport was detected regarding this nutrient in 7% of the participants. On the other hand, 20% of women presented an underreport of the potassium intake. Means of this mineral intake obtained by the biomarker and the 24h-R differ in less than 1% for men, but in more than 11% for women. The underreport of sodium intake was identified in almost 35% of the individuals, with a mean magnitude of 38%, regardless of gender. The 24h-R correlation of means with the respective biomarkers was 0.58 for proteins, 0.31 for potassium, and 0.30 for sodium. The general data from this study still wait for the conclusion of the doubly labeled water analyses, which will permit the evaluation of the energy intake underreport.

### Nutritional Composition Changes

When evaluated the implication of the change of the nutritional composition table compiled and previously published by IBGE^11^ and TBCA 7.0, some crucial variations were found mainly regarding micronutrients. Table 1 presents this comparison in two perspectives: 1) means of populational intake obtained from the IBGE and TBCA tables to evaluate the extent of differences identified while estimating nutrient intake in the population; 2) means of composition for each nutrient per 100 grams of food to identify the nutrients with more differences between the tables. This comparison was made for the food intake data obtained in INA 2008−2009. Generally, the mean variation of energy, macronutrients, and minerals was relatively low (less than 15% between the tables), except for trans fats and selenium, with means 40% and 52% lower in TBCA. For vitamins, except for vitamin E that had a positive variation, the mean content in TBCA varied between -11% and -60% compared with the table adopted in INA 2008–2009. Most of these differences impacted the estimation of intake means in the population: lower means for trans fats, selenium, sodium, thiamine, riboflavin, pyridoxine, and vitamins C and D, which varied between -20% and -60% using TBCA compared with the estimations of the IBGE table. On the other hand, the intake mean with TBCA was 20% higher for polyunsaturated fats and fibers, and 40% higher for vitamin E.

**Table 1:** Intake and composition means in 100 grams of a piece of food regarding energy, macro and micronutrients estimation based on the composition tables of IBGE and TBCA, data from the National Dietary Survey, 2008–2009.

Nutrients	Mean intake				Mean composition			
	Table IBGE		TBCA		Table IBGE		TBCA	
	Mean	SD	Mean	SD	Mean	SD	Mean	SD
Energy (kcal)	1,778.0	806.7	1,742.7	778.5	195.1	133.0	195.3	136.5
Proteins (g)	82.0	47.5	83.0	46.3	13.6	12.0	13.0	11.3
Total fats (g)	55.7	33.3	57.4	33.0	9.3	11.3	9.5	11.4
Saturated fat (g)	19.3	13.5	19.7	12.7	3.2	4.7	3.3	4.6
Monounsaturated fat (g)	18.8	12.8	18.0	11.0	3.4	4.9	3.4	4.8
Polyunsaturated fats (g)	11.4	7.3	13.8	9.1	1.7	3.0	1.9	3.5
Trans fats (g)	2.2	2.8	1.4	1.7	0.3	0.8	0.2	0.4
Carbohydrates (g)	235.1	113.5	233.6	109.1	14.1	22.0	15.0	22.0
Fibers (g)	20.5	11.6	24.7	15.1	1.3	3.1	1.5	3.2
Calcium (mg)	489.4	351.9	437.5	359.3	51.4	117.9	54.0	124.3
Magnesium (mg)	244.1	133.6	273.6	134.8	25.9	41.8	26.2	50.1
Phosphorus (mg)	978.2	567.1	1,030.7	523.1	138.3	124.4	135.9	128.4
Iron (mg)	11.4	6.2	11.4	5.8	1.8	3.2	1.7	3.4
Sodium (mg)^b^	3,172.0	2,293.1	2,477.9	1,389.2	356.7	570.2	316.5	415.2
Potassium (mg)	2,367.5	1,172.7	2,328.7	1,084.9	230.4	207.3	233.0	212.8
Copper (mg)	1.3	2.9	1.5	2.5	0.2	1.2	0.2	1.0
Zinc (mg)	11.3	7.2	11.6	7.1	2.0	6.0	1.9	2.8
Selenium (mg)	91.4	89.3	44.4	56.2	18.4	62.0	8.8	75.1
Vitamin A (mcg RAE)	510.9	1,926.0	525,6	2,751.6	167.2	908.2	138.6	1,099.6
Thiamine (mg)	1.2	0.7	0.9	0.7	0.1	0.3	0.1	0.2
Riboflavin (mg)	1.6	1.0	1.2	0.9	0.2	0.4	0.1	0.3
Pyridoxine (mg)	1.4	0.8	0.7	0.6	0.2	0.2	0.1	0.2
Vitamin C (mg)	219.1	933.8	134.8	242.2	8.8	74.3	7.0	49.7
Vitamin D (mg)	3.6	6.3	2.1	3.4	0.4	1.0	0.3	0.8
Vitamin E (mg)	4.0	2.5	6.6	7.5	0.6	1.7	0.9	2.3
Cobalamin (mcg)	5.5	14.5	5.8	13.1	2.0	8.7	1.4	5.4
Folate (mcg DFE)	395.7	242.4	441.7	248.7	35.7	96.5	27.8	54.0

a Mean composition of 100g of a piece of food;

b intrinsic sodium of food + sodium added during preparation.

### Data-Collecting Changes

To evaluate and to prevent artificial variations in the analysis of nutrient intake evolution, the data of 2008–2009 were re-analyzed using TBCA 7.0, consideringthe differences with the former IBGE composition table regarding the origin of data, the methods of recipes nutritional calculation, and the food codification quality^11–13^. When TBCA was applied for INA 2008–2009, the change of the term cooked meat for braised meat was ignored, as done in the former analyses, since this procedure presented no impact in the estimations of energy and lipids intake. The means of energy intake before and after the substitution of the type of meat preparation was similar, presenting only a small difference in confidence intervals, being equal to 1,753 kcal (CI95%: 1,734–1,772) and 1,753 kcal (CI95%: 1,733–1,771), respectively. Table 2 presents the mean of added sugar intake in both INA, using different analysis strategies. As expected, the addition sugar in beverages based on the previously cited question increased the mean of added sugar intake, presenting results more than double in adult and old age participants. However, INA 2017–2018 compared means of sugar intake using two different strategies: 1) a generic question about the frequency of sugar consumption, and 2) the amount of added sugar reported. The results were lower for the second strategy, although the confidence intervals only overlapped for adults of both genders.

**Table 2:** Mean (CI95%) of added sugar intake (g) in the first day of food consumption according to gender and age in the Brazilian National Dietary Surveys (INA) 2008–2009 and 2017–2018, using evaluation strategies of added sugar.

		Added sugar (g)							
		INA 2008–2009^a^		INA 2008–2009^b^		INA 2017–2018^b^		INA 2017–2018^c^	
**Men**									
	Teenager	45.2	(41.9–48.9)	76.0	(72.5–79.5)	62.8	(60.1–65.6)	59.4	(56.5–62.2)
	Adult	30.2	(28.7–31.6)	63.2	(61.5–64.9)	51.8	(50.3–53.2)	47.3	(45.8–48.7)
	Old age	19.9	(17.7–21.1)	48.4	(45.2–51.6)	39.3	(37.3–41.2)	35.8	(33.8–37.8)
**Women**									
	Teenager	46.2	(43.2–49.8)	74.8	(71.5–78.1)	56.1	(53.7–58.5)	51.9	(49.5–54.4)
	Adult	27.4	(26.2–28.7)	57.7	(56.2–59.1)	44.1	(43.1–45.2)	40.4	(39.4–41.5)
	Old age	19.5	(17.8–21.1)	42.4	(40.0–44.9)	35.5	(33.8–37.2)	32.9	(31.1–34.7)

a There was no strategy to analyze added sugar, considering only the added sugar mean, disregarding the generical question about added sugar on beverages.

b The question about the frequency of added sugar was considered as 10% of the beverage volume for individuals that reported using only sugar as a sweetener and 5% for those who reported sugar and a sweetener. The report of added sugar as an added item in INA 2017–2018 was ignored.

c Only the report of added sugar as an added item was considered.

## DISCUSSION

This study detailed the methodological differences of both INA, the first in 2008–2009 and the second in 2018-2018, conducted in Brazil and their possible impact in the comparison. Important alterations occurred: the data-collecting instrument changed and the energy, macro and micronutrients intake became based on TBCA. This is adequate for the food consumed in Brazil and is certified by FAO. Nevertheless, the alteration affected only slightly the mean composition of the components evaluated for energy, macronutrients, and minerals intake, presenting more significant differences for vitamins.

The validation study data of the INA 2017–2018 – although incomplete by the absence of the doubly labeled water results – indicate underreport of protein and sodium, and higher percentage of underreport for potassium in women than in men, with values around 20%. Other international^14^ and national^15^ analyses found similar results for proteins. Underreport in all studies is the major limitation for recalls or reports to evaluate food intake.

Regarding the energy intake mean found in the recalls, the values are higher than those obtained in the validation study of the instrument used in 2008–2009, which presented values of 2,017 kcal for men, SD = 548, and 1,611 kcal for women, SD = 4526. Thus, the underreport in 2017–2018 was probably lower, as the values for men and women were 2,339 kcal and 1,863 kcal, using the same age ranges in both studies.

The detailed analysis of fats and sugars concerning the changes in methods and strategies of analysis to determine them in both surveys indicated a low variation for fats, but a significant variation for added sugar.

The strategy of considering the oil addition in cooked meat, which probably would increase the percentage of fats and energy, presented no impact on the means. Possible explanations for such findings are the differences in yield factors, such as the change of food weight after cooking and the different types of oil addition used to measure the recipe composition. These factors can in part explain the variations in energy density of braised meat between the table of 2008–2009, based on the software NDSR, and the TBCA^16^. Besides, cooked and braised meats in TBCA differ slightly regarding added oil, approximately 1 g of soy oil for 100 g of a piece of food, which may have contributed for the results observed^12^.

Concerning added sugar, it is worth mentioning that the intention of using a generic question to assess the amount of sugar to sweeten beverages was to recuperate data about individuals’ sugar intake as no question regarded the use of sugar to sweeten specific types of food and beverage. The results found are relevant to interpret the findings of both surveys. Comparing the estimations of INA 2008–2009 – which omitted the added sugar on juices – with those of INA 2017–2018, an increase of sugar intake occurred. On the other hand, comparing the estimations of INA 2008–2009 with the question about the frequency of sugar consumption, sugar intake reduced in the decade analyzed. This interpretation completely distinct from the results shows how much the change of analysis strategies for food intake may induce to conflicting conclusions. not using sugar neither

A study by IBGE^3^ compared the means of sugar intake without considering the generic question about sugar intake in beverages and the means found in 2017–2018. Sugar was included as an added item, and the calculation of means included this value. Comparing the results found in INA 2017–2018, Brazilians’ frequency of sugar to sweeten beverage or food dropped from 90.8% in 2008–2009 to 85.4% in 2017–2018. On the other hand, not using sugar neither sweetener increased from 1.6% to 6.1%, and the use of sweeteners increased from 7.6% to 8.5% in the same period^2,3^. There was also a reduction of soda drinks and commercial juices consumption (beverages that significantly increase added sugar intake) in both genders and all age and income ranges between both surveys^3^. Availability data also follow this reduction pattern^17^.

Such results are coherent with the reduction of sugar intake in the period studied. They reiterate that the most appropriate comparison between both surveys would consider INA 2008–2009 data with the added sugar on beverages, using the frequency of sugar consumption. Besides, it is believed that sugar intake estimations as an added item in specific types of food are closer to the real use compared with the generic question. Thus, comparing the evolution of added sugar intake between both surveys is recommended, using INA 2008–2009 data with the generic question about the most frequent method of sweetening, and INA 2017–2018 data considering added sugar.

Generally, the methodological changes included in INA enhanced the estimation of food groups and nutrients intake. Besides, such alterations detailed and specified data regarding food intake reports, typifying and detailing meals taken outside the household, which may contribute for a better comprehension and analysis of Brazilian’s nutrition.
