# Supplementary material for: Limitations in the comparison of the Brazilian National Dietary Surveys of 2008–2009 and 2017–2018
Source: Rev Saude Publica. 2021 Nov 22;55 Supl 1:3s. doi: 10.11606/s1518-8787.2021055003365 (PMC9586432; doi:10.11606/s1518-8787.2021055003365)
Supplement: Translation

# Translation: Limitações na comparação dos Inquéritos Nacionais de Alimentação de 2008–2009 e 2017–2018

**Authors:** Renata Muniz Rodrigues, Eduardo De Carli, Marina Campos Araújo, Eliseu Verly, Dirce Maria Lobo Marchioni, Ilana Nogueira Bezerra, Amanda de Moura Souza, Edna Massae Yokoo, Rosangela Alves Pereira, Rosely Sichieri

**Affiliations:** I Rio de Janeiro RJ Brasil Universidade do Estado do Rio de Janeiro. Instituto de Medicina Social. Rio de Janeiro, RJ, Brasil; II São Paulo SP Brasil Universidade de São Paulo. Faculdade de Saúde Pública. Departamento de Nutrição. São Paulo, SP, Brasil; III Rio de Janeiro RJ Brasil Fundação Oswaldo Cruz. Escola Nacional de Saúde Pública Sergio Arouca. Departamento de Epidemiologia e Métodos Quantitativos em Saúde. Rio de Janeiro, RJ, Brasil; IV Fortaleza CE Brasil Universidade Estadual do Ceará. Centro de Ciências da Saúde. Fortaleza, CE, Brasil.; V Rio de Janeiro RJ Brasil Universidade Federal do Rio de Janeiro. Instituto de Estudos em Saúde Coletiva. Rio de Janeiro, RJ, Brasil; VI Niterói RJ Brasil Universidade Federal Fluminense. Departamento de Epidemiologia e Bioestatística. Niterói, RJ, Brasil; VII Rio de Janeiro RJ Brasil Universidade Federal do Rio de Janeiro. Departamento de Nutrição Social e Aplicada. Rio de Janeiro, RJ, Brasil

**Keywords:** Inquéritos sobre Dietas, métodos, Coleta de Dados, Tabela de Composição de Alimentos, Utilização de Procedimentos e Técnicas

## Abstract

**OBJETIVO:**

Apresentar particularidades dos dois Inquéritos Nacionais de Alimentação (INA) e o processo metodológico empregado para comparação dos dados.

**MÉTODOS:**

O estudo detalha as diferenças entre os INA realizados pelo IBGE em 2008–2009 e em 2017–2018, apresentando as alterações nos métodos de coleta de dados e nas tabelas de composição dos alimentos, assim como as estratégias de análise recomendadas para comparação dos dados. Um estudo de validação foi realizado com 95 participantes da terceira onda do Estudo Longitudinal de Saúde do Adulto a fim de avaliar o erro de medição associado aos procedimentos adotados nos recordatórios de 24 horas do INA 2017–2018. Empregaram-se biomarcadores de recuperação urinária de proteínas, sódio e potássio como padrões de referência. Na análise dos INA, diferentes estratégias foram elaboradas para comparar dois itens importantes do consumo alimentar que sofreram mudanças na forma de coleta: as gorduras e os açúcares.

**RESULTADOS:**

O estudo de validação do instrumento indicou menor sub-relato no inquérito mais recente, com maiores médias de ingestão de energia. A correlação das medidas dos recordatórios de 24 horas com os respectivos biomarcadores foi de 0,58 para proteínas, 0,31 para potássio e 0,30 para sódio. Comparando as tabelas de composição utilizadas nos dois inquéritos com os dados obtidos no INA 2008–2009, a variação média de energia, macronutrientes e minerais foi menor que 15%, com exceção das gorduras trans e selênio, com médias 40% e 52% menores na TBCA. No INA 2017–2018, as médias do consumo de açúcar de adição foram menores, usando a informação do açúcar reportado como item de adição comparada com a pergunta genérica sobre a frequência do uso do açúcar.

**CONCLUSÃO:**

As mudanças metodológicas incluídas no INA atual permitiram aprimorar as estimativas de consumo de grupos de alimentos e nutrientes, acrescentando informações mais detalhadas e específicas aos relatos do consumo alimentar.

## INTRODUÇÃO

No Brasil, até 2008, as estimativas de consumo alimentar e de mudanças temporais eram feitas a partir de dados de disponibilidade domiciliar de alimentos obtidos nas Pesquisas de Orçamentos Familiares (POF), realizadas periodicamente desde a década de 1970 pelo Instituto Brasileiro de Geografia e Estatística (IBGE). Essas estimativas são realizadas com base em dados de agregados, como setores censitários ou estratos amostrais, e portanto são indiretas. Além disso, a informação sobre disponibilidade domiciliar não considera a alimentação fora do domicílio, o que leva à subestimação do consumo^1^. Embora as estimativas agregadas permitam comparações nacionais e internacionais, até a primeira década do século XXI o país não contava com informações sobre consumo alimentar individual de representatividade nacional que permitissem compreender características desse consumo segundo faixa etária, sexo e outras características individuais.

Nas POFs de 2008–2009 e 2017–2018 foi incluído um módulo, intitulado Inquérito Nacional de Alimentação (INA), que obteve dados de consumo alimentar para todos os indivíduos com pelo menos 10 anos de idade em uma subamostra probabilística dos domicílios selecionados para a amostra original da POF. Em 2008–2009, foram estudados 13.569 domicílios, que corresponderam a 24,3% da amostra geral da POF^2^. Em 2017–2018, a proporção de domicílios que compuseram a subamostra foi de 34,7%, tendo sido investigados 20.112 domicílios^3^. Ambos os inquéritos permitem estimativas para as regiões do país (Norte, Nordeste, Sudeste, Sul e Centro-Oeste) e por situação urbana e rural.

Diferentemente do que ocorre com dados de disponibilidade domiciliar de alimentos, nos inquéritos foi possível estimar a alimentação fora do domicílio e caracterizá-la segundo os grupos de alimentos consumidos e padrões alimentares, bem como estimar a ingestão de energia e nutrientes e a inadequação da ingestão de micronutrientes. Como ocorreram mudanças nos métodos de coleta de dados e nas tabelas de composição centesimal dos alimentos, o presente estudo tem como objetivo apresentar as particularidades dos dois INA e uma metodologia que permite a comparação dos dados, visando analisar a evolução do consumo alimentar.

## MÉTODOS

### Instrumentos de Coleta

Nos dois inquéritos, o consumo de alimentos e bebidas foi avaliado em dois dias não consecutivos, escolhidos ao longo da semana em que o agente de pesquisa esteve no domicílio. Foram avaliados todos os moradores do domicílio com 10 anos ou mais de idade, de tal forma que todos os dias da semana fossem representados.

O instrumento utilizado em 2008–2009 foi o registro alimentar. Os indivíduos eram orientados a preencher as informações no mesmo momento que realizavam suas refeições, detalhando os alimentos e bebidas consumidos, a forma de preparação, a unidade de medida utilizada, a quantidade consumida, o horário e o local do consumo. Todos os alimentos e bebidas ingeridos nos dias indicados deviam ser registrados. As anotações eram revisadas pelo agente de pesquisa e passadas para o sistema computadorizado de entrada de dados, no próprio domicílio. Nos casos em que havia impedimento para algum morador preencher o registro, outro morador poderia ajudá-lo.

Em 2017–2018 utilizou-se como instrumento de coleta o recordatório de 24 horas (R24h). Os indivíduos foram indagados, em entrevistas pessoais, sobre todos os alimentos e bebidas (incluindo água) consumidos no dia anterior a cada uma das duas entrevistas. A entrevista foi realizada utilizando um *software* elaborado especificamente para a pesquisa, desenvolvido para *tablet*, seguindo um roteiro estruturado, em estágios sequenciais de questionamento da alimentação, com base no método automatizado de múltiplas passagens^4^. As informações, à medida que eram relatadas sem interrupção pelo informante, levavam à elaboração de uma lista rápida, anotada em papel, incluindo horários e ocasiões de consumo. A partir dessa lista rápida, o agente de pesquisa registrava os dados no *software*, detalhando as informações relativas aos alimentos e bebidas consumidas.

A opção por adotar o R24h se deveu, primeiramente, por esse instrumento ser utilizado quase universalmente em pesquisas de base populacional, sendo considerado o método com menor possibilidade de erro sistemático^5^. Outro motivo foram os resultados do estudo de validação do instrumento aplicado em 2008–2009, que indicaram melhor desempenho do recordatório quando comparado ao registro alimentar, utilizando como método padrão-ouro a água duplamente marcada^6^. No Quadro 1 são detalhadas as mudanças no método de coleta de dados entre os dois inquéritos.

**Quadro 1:** Quadro-resumo dos instrumentos de coleta dos Inquéritos Nacionais de Alimentação, 2008–2009 e 2017–2018.

	INA 2008–2009	INA 2017–2018
**Instrumento de coleta**	Registro alimentar	Recordatório de 24 horas
**Itens alimentares**	1.503	1.832
**Local do consumo**	Dentro ou fora do domicílio	Em casa ou levado de casa. Merenda escolar.
		Restaurante por quilo. Cantina, bar, lanchonete, *fast food*.
		Restaurante – outros.
		Vendedores ambulantes ou de rua. Fora de casa – outros.
**Formas de preparação**	15 opções	9 opções
**Unidades de medidas caseiras**	106 opções (incluindo g, kg, ml e l)	64 opções
**Ocasião de consumo**	Não existia	Café da manhã; almoço; lanche; jantar; ceia; outra ocasião.
**Itens de adição**	Não existia	12 itens: azeite, manteiga/margarina, açúcar, adoçante, mel, melado, maionese, ketchup, mostarda, molho shoyu, queijo ralado e creme de leite.

Tanto em 2008–2009 quanto em 2017–2018, os programas de entrada de dados computadorizados alertavam quando o indivíduo relatava menos de cinco itens alimentares ou quando se verificavam mais de três horas sem relato de nenhum item alimentar. Nesses casos, o agente de pesquisa deveria confirmar se todos os alimentos consumidos nos dias pesquisados foram relatados e investigar possíveis omissões ou dados incompletos.

A base de dados de alimentos e bebidas foi de 1.503 itens em 2008–2009 e de 1.832 itens em 2017–2018. A nova listagem de alimentos foi atualizada a partir dos dados do Estudo de Riscos Cardiovasculares em Adolescentes^7^ e dos inquéritos realizados para atualização do estudo GloboDiet^8,9^.

Nos dois inquéritos, para cada alimento ou bebida relatado, o *software* requeria a informação da quantidade consumida (unidade de medida e quantidade). Em 2017–2018, para elaborar a base de dados das quantidades em gramas ou mililitros, realizou-se uma extensa revisão e atualização da Tabela de Medidas Referidas para os Alimentos Consumidos no Brasil da POF 2008–2009^10^. Em 2008–2009, o *software* permitia o registro de variações de quilogramas e litro em 106 unidades de medida. Em 2017–2018, estavam disponíveis 64 unidades de medida. Maiores detalhes são encontrados nas publicações que relatam os dois inquéritos^3,10^.

Em ambos os inquéritos, eram solicitadas informações sobre local e horário do consumo, de 0 a 23 horas, sem casas decimais. Em 2008–2009, para local de consumo, considerou-se “Dentro” quando o alimento consumido era proveniente do domicílio e “Fora” quando o alimento era adquirido e consumido fora do domicílio. Em 2017–2018, para registrar o local de consumo foram disponibilizadas as opções domicílio, trabalho, escola, restaurantes, cantinas, vendedores ambulantes e outros locais fora de casa.

Para alimentos selecionados, especialmente hortaliças e carnes, solicitava–se o detalhamento da forma de preparação. Em 2008–2009 o *software* apresentava 15 opções de forma de preparação; em 2017–2018, 9 opções. A redução se deu porque preparações como à milanesa, com molho branco ou vermelho, ao alho e óleo, mingau e sopa foram incorporadas aos itens alimentares correspondentes. Para alimentos específicos, para os quais não são esperadas variações na forma de preparação, como pão, leite e iogurte, utilizou-se a opção “não se aplica” na forma de preparação, em ambos os inquéritos.

O inquérito de 2017–2018 inovou ao solicitar que o informante nomeasse a ocasião de consumo e itens de adição. Para designar a ocasião de consumo, foram disponibilizadas as opções “café da manhã”, “almoço”, “lanche”, “jantar”, “ceia” e “outra ocasião”. Em 2008–2009, possíveis adições eram relatadas como alimentos, mas em 2017–2018 uma lista de 12 itens estava disponível para registrar a adição de itens usualmente consumidos juntos, por exemplo, manteiga ou margarina com pão, milho e biscoitos, ou mostarda com sanduíches, saladas e batatas. As opções de adição eram específicas para cada item alimentar. Quando apropriado, as opções de resposta para cada item de adição eram “sim”, “não” ou “não se aplica”. O indivíduo poderia relatar quantas adições quisesse para cada item alimentar, de acordo com seu consumo. Não foi coletada a quantidade usada de cada item de adição.

### Validade dos Instrumentos

Como já referido, em 2008–2009 um estudo de validação realizado em uma amostra de conveniência, com análises de água duplamente marcada, verificou sub–relato da ingestão de energia da ordem de 30%^6^. Para o inquérito de 2017–2018, a fim de avaliar erros de medição associados aos procedimentos adotados nos R24h, um estudo de validação foi realizado empregando biomarcadores de recuperação urinária de proteínas (nitrogênio), sódio e potássio como padrões de referência.

Foram investigados 95 indivíduos adultos e idosos, com idades entre 44 e 72 anos, 54,2% de mulheres, participantes da terceira onda do Estudo Longitudinal de Saúde do Adulto em São Paulo (SP). Esses indivíduos referiram estabilidade do peso corporal nos últimos seis meses e nenhum registro de doenças ou uso contínuo de medicamentos e/ou suplementos vitamínico-minerais. Médias das ingestões diárias de proteína, sódio e potássio, ajustadas pela variabilidade intrapessoal, foram calculadas a partir de dois R24h e duas amostras de urina de 24 horas. Biomarcadores de recuperação urinária foram obtidos de 83 participantes.

### Dados de Composição de Alimentos

Em 2008–2009, para as estimativas de ingestão de energia e nutrientes, elaborou-se uma tabela de composição dos alimentos citados no INA^11^ a partir de compilação de informações reconhecidas, como como a Tabela Brasileira de Composição de Alimentos - TACO (NEPA, 2006) e a base de dados do Nutrition Data System for Research (NDSR), da Universidade de Minnesota. Em 2017–2018, as estimativas de ingestão de nutrientes foram calculadas com o auxílio da versão 7.0 da TBCA, elaborada pela Rede Brasileira de Dados de Composição de Alimentos, Universidade de São Paulo e Centro de Pesquisa em Alimentos, disponível em http://www.tbca.net.br/^12^.

A TBCA é adequada ao contexto dos inquéritos nacionais e tem sua qualidade e confiabilidade asseguradas por diretrizes e critérios para geração, compilação e utilização de dados de composição de alimentos estabelecidos pela International Network of Food Data Systems, entidade da Organização das Nações Unidas para a Alimentação e a Agricultura (FAO)^12,13^.

As diferenças entre os inquéritos de 2008–2009 e 2017–2018, expressas nas mudanças nos métodos de avaliação e na utilização de nova tabela de composição de alimentos, implicaram desafios técnicos para a comparação de dois itens importantes do consumo alimentar: as gorduras e os açúcares. Na primeira versão do INA, a composição nutricional de carnes cozidas não contemplava nenhum tipo de gordura na forma de preparo^11^. Entretanto, assumindo que, tradicionalmente, indivíduos que relatam o consumo de carnes cozidas utilizam algum tipo de gordura na preparação, optou–se por substituir, por meio de rotinas de programação, a forma de preparação cozida para refogada, visando aproximar a composição nutricional desses alimentos quando preparados com óleo^2^. No INA 2017–2018, as formas de preparação foram revistas, e a preparação cozida foi subdividida em com ou sem gordura. Nesse caso, foi possível identificar os indivíduos que utilizaram ou não gordura no cozimento das carnes^3^.

A análise da evolução do consumo de açúcar foi tecnicamente mais desafiadora. Nos dois inquéritos foi incluída uma pergunta genérica e qualitativa sobre a forma de adoçar utilizada com mais frequência em alimentos e bebidas (açúcar e/ou adoçante, ambos, ou nenhum dos dois), mas sem especificar os itens alimentares aos quais o açúcar costumava ser adicionado. No INA 2008–2009, essas respostas foram consideradas na análise dos dados de energia e nutrientes, sendo que a adição de açúcar na proporção de 10% do volume das bebidas foi adotada para indivíduos que relataram utilizar exclusivamente açúcar, e 5% para aqueles que relataram utilizar ambos, açúcar e adoçante^11^. Já no INA 2017–2018, o açúcar foi incluído entre as 12 opções de adições possíveis a cada item alimentar. Essa questão foi também qualitativa, e um percentual padrão de 10% foi estabelecido para adição do açúcar à quantidade relatada do alimento ou bebida^3^.

## RESULTADOS

### Estudo de Validação

Para os 95 participantes do estudo, a média de ingestão de energia avaliada pelos dois recordatórios foi de 2.078 kcal, com desvio-padrão (DP) de 663. Esse valor foi de 2.339 kcal para os homens (n = 43), com DP = 691, e 1.863 kcal para as mulheres (n = 52), com DP = 560.

Os resultados de biomarcadores, utilizando a mesma base do *software* do INA 2017–2018, indicaram variação nos erros de medidas dietéticas entre os nutrientes e os sexos. Para a proteína, a média dos R24h não diferiu significantemente da obtida com o biomarcador, ainda que tenha se detectado sub-relato desse nutriente em 7% dos participantes. Por outro lado, houve sub-relato da ingestão de potássio em 20% das mulheres. Médias da ingestão desse mineral, obtidas pelo biomarcador e pelos R24h, diferiram em menos 1% para os homens, mas em mais de 11% para as mulheres. O sub-relato da ingestão de sódio foi identificado em aproximadamente 35% dos indivíduos, com magnitude média de 38%, independentemente de sexo. A correlação das medidas dos R24h, com os respectivos biomarcadores, foi de 0,58 para proteínas, 0,31 para potássio e 0,30 para sódio. Os dados gerais do estudo aguardam a finalização das análises de água duplamente marcada, que permitirá avaliar a ocorrência de sub-relato de energia.

### Mudanças na Composição Nutricional

Quando avaliadas as implicações da mudança da tabela de composição nutricional compilada e publicada anteriormente pelo IBGE^11^ pela TBCA 7.0, foram encontradas algumas variações importantes, principalmente em relação aos micronutrientes. A Tabela 1 traz essa comparação em duas perspectivas: 1) médias de ingestão populacional obtidas a partir das tabelas IBGE e TBCA, para avaliar o peso das divergências identificadas na estimativa da ingestão de nutrientes na população; 2) médias de composição de cada nutriente por 100 gramas dos alimentos, para identificar os nutrientes com maiores divergências entre as tabelas. Essa comparação foi feita para os dados de consumo alimentar obtidos no INA 2008−2009. De forma geral, a variação média de energia, macronutrientes e minerais foi relativamente baixa (menos de 15% entre as tabelas), com exceção das gorduras trans e selênio, com médias 40% e 52% menores na TBCA. Para as vitaminas, com exceção da vitamina E, que teve variação positiva, o conteúdo médio na TBCA variou de -11% a -60% em relação à tabela adotada no INA 2008–2009. A maioria dessas diferenças teve impacto nas estimativas das médias de ingestão na população: médias inferiores para gorduras trans, selênio, sódio, tiamina, riboflavina, piridoxina e vitaminas C e D, que variam de -20% a -60% utilizando a TBCA em relação às estimativas a partir da tabela do IBGE. Por outro lado, com a TBCA, a média de ingestão foi 20% maior para gordura poli-insaturada e fibras, e 40% maior para vitamina E.

**Tabela 1:** Médias de ingestão e de composição em 100 gramas de alimentos para energia, macro e micronutrientes estimadas a partir da tabela de composição do IBGE e TBCA, dados do Inquérito Nacional de Alimentação, 2008–2009.

Nutrientes	Ingestão média				Composição média^a^			
	Tabela IBGE		TBCA		Tabela IBGE		TBCA	
	Média	DP	Média	DP	Média	DP	Média	DP
Energia (kcal)	1.778,0	806,7	1.742,7	778,5	195,1	133,0	195,3	136,5
Proteínas (g)	82,0	47,5	83,0	46,3	13,6	12,0	13,0	11,3
Gorduras totais (g)	55,7	33,3	57,4	33,0	9,3	11,3	9,5	11,4
Gordura saturada (g)	19,3	13,5	19,7	12,7	3,2	4,7	3,3	4,6
Gord. monoinsaturada (g)	18,8	12,8	18,0	11,0	3,4	4,9	3,4	4,8
Gord. poli-insaturada (g)	11,4	7,3	13,8	9,1	1,7	3,0	1,9	3,5
Gorduras trans (g)	2,2	2,8	1,4	1,7	0,3	0,8	0,2	0,4
Carboidratos (g)	235,1	113,5	233,6	109,1	14,1	22,0	15,0	22,0
Fibras (g)	20,5	11,6	24,7	15,1	1,3	3,1	1,5	3,2
Cálcio (mg)	489,4	351,9	437,5	359,3	51,4	117,9	54,0	124,3
Magnésio (mg)	244,1	133,6	273,6	134,8	25,9	41,8	26,2	50,1
Fósforo (mg)	978,2	567,1	1.030,7	523,1	138,3	124,4	135,9	128,4
Ferro (mg)	11,4	6,2	11,4	5,8	1,8	3,2	1,7	3,4
Sódio (mg)^b^	3.172,0	2.293,1	2.477,9	1.389,2	356,7	570,2	316,5	415,2
Potássio (mg)	2.367,5	1.172,7	2.328,7	1.084,9	230,4	207,3	233,0	212,8
Cobre (mg)	1,3	2,9	1,5	2,5	0,2	1,2	0,2	1,0
Zinco (mg)	11,3	7,2	11,6	7,1	2,0	6,0	1,9	2,8
Selênio (mg)	91,4	89,3	44,4	56,2	18,4	62,0	8,8	75,1
Vitamina A (mcg RAE)	510,9	1.926,0	525,6	2.751,6	167,2	908,2	138,6	1.099,6
Tiamina (mg)	1,2	0,7	0,9	0,7	0,1	0,3	0,1	0,2
Riboflavina (mg)	1,6	1,0	1,2	0,9	0,2	0,4	0,1	0,3
Piridoxina (mg)	1,4	0,8	0,7	0,6	0,2	0,2	0,1	0,2
Vitamina C (mg)	219,1	933,8	134,8	242,2	8,8	74,3	7,0	49,7
Vitamina D (mg)	3,6	6,3	2,1	3,4	0,4	1,0	0,3	0,8
Vitamina E (mg)	4,0	2,5	6,6	7,5	0,6	1,7	0,9	2,3
Cobalamina (mcg)	5,5	14,5	5,8	13,1	2,0	8,7	1,4	5,4
Folato (mcg DFE)	395,7	242,4	441,7	248,7	35,7	96,5	27,8	54,0

a Composição média por 100g do alimento;

b sódio intrínseco dos alimentos + sódio adicionado nas preparações.

### Mudanças no Método de Coleta

Para avaliar e posteriormente prevenir variações artificiais na análise da evolução da ingestão de nutrientes, os dados de 2008–2009 foram reanalisados com a TBCA 7.0, considerando suas divergências com a antiga tabela de composição do IBGE em relação à origem dos dados, aos métodos para o cálculo nutricional de receitas e à qualidade da codificação dos alimentos^11–13^. Quando a TBCA foi aplicada ao INA 2008–2009, optou–se por não realizar a mudança da preparação de carnes cozidas para refogadas, procedimento utilizado nas análises de dados anteriores, uma vez que esse procedimento não mostrou impacto nas estimativas de ingestão de energia e lipídios. As médias do consumo de energia antes e após a substituição da forma de preparação das carnes foram similares, com pequena diferença somente nos intervalos de confiança, sendo iguais a 1.753 kcal (IC95%: 1.734–1.772) e 1.753 kcal (IC95%: 1.733–1.771), respectivamente.

A Tabela 2 apresenta a média de ingestão do açúcar de adição nos dois INA utilizando diferentes estratégias de análise. Como esperado, com o acréscimo de açúcar nas bebidas baseado na questão citada, a média de consumo de açúcar de adição foi mais elevada do que a obtida quando essa informação não foi considerada, diferindo em mais do que o dobro para adultos e idosos. Já no INA 2017–2018 compararam-se as médias do consumo de açúcar utilizando duas estratégias diferentes: 1) a pergunta genérica sobre a frequência do uso do açúcar e 2) o açúcar reportado como item de adição. Os resultados foram menores para a segunda estratégia, embora os intervalos de confiança só não tenham se sobreposto para adultos de ambos os sexos.

**Tabela 2:** Média (IC95%) da ingestão de açúcar de adição (g) do primeiro dia de consumo alimentar de acordo com sexo e idade dos Inquéritos Nacionais de Alimentação (INA) 2008–2009 e 2017–2018 utilizando estratégias de avaliação do açúcar de adição.

		Açúcar de adição (g)							
		INA 2008–2009^a^		INA 2008–2009^b^		INA 2017–2018^b^		INA 2017–2018^c^	
**Homens**									
	Adolescente	45,2	(41,9–48,9)	76,0	(72,5–79,5)	62,8	(60,1–65,6)	59,4	(56,5–62,2)
	Adulto	30,2	(28,7–31,6)	63,2	(61,5–64,9)	51,8	(50,3–53,2)	47,3	(45,8–48,7)
	Idoso	19,9	(17,7–21,1)	48,4	(45,2–51,6)	39,3	(37,3–41,2)	35,8	(33,8–37,8)
**Mulheres**									
	Adolescente	46,2	(43,2–49,8)	74,8	(71,5–78,1)	56,1	(53,7–58,5)	51,9	(49,5–54,4)
	Adulto	27,4	(26,2–28,7)	57,7	(56,2–59,1)	44,1	(43,1–45,2)	40,4	(39,4–41,5)
	Idoso	19,5	(17,8–21,1)	42,4	(40,0–44,9)	35,5	(33,8–37,2)	32,9	(31,1–34,7)

a Não houve nenhuma estratégia de análise para o açúcar de adição, analisando-se apenas a média de açúcar de adição, sem considerar a pergunta genérica de adição de açúcar nas bebidas.

b Foi considerada a pergunta sobre a frequência do uso de açúcar para adição de 10% do volume de bebidas para os indivíduos que relataram utilizar exclusivamente açúcar para adoçar e 5% para aqueles que relataram utilizar açúcar e adoçante. O relato de açúcar de adição como ingrediente de adição no INA 2017–2018 foi desconsiderado.

c Foi considerado somente o relato de açúcar de adição como ingrediente de adição.

## DISCUSSÃO

O presente estudo detalhou as diferenças nos métodos utilizados nos dois INA realizados no Brasil, o primeiro em 2008–2009 e o segundo em 2017–2018, e seu possível impacto nas comparações. Importantes alterações ocorreram: o instrumento de coleta foi alterado e a avaliação do consumo de energia, macro e micronutrientes baseou-se na TBCA, que é mais adequada aos alimentos consumidos no Brasil e assegurada pela FAO. Verificou-se, contudo, que a mudança pouco afetou a composição média dos componentes analisados para energia, macronutrientes e minerais, mas diferenças acentuadas ocorreram para as vitaminas.

Os dados do estudo de validação para o INA 2017–2018 – ainda que incompletos, por conta da ausência de resultados da água duplamente marcada – indicam sub-relato de proteína e sódio, com maiores percentuais de sub-relato de potássio entre mulheres do que entre homens, com valores ao redor de 20%. Resultados semelhantes para proteínas foram encontrados em outras análises, tanto internacionais^14^ como nacionais^15^, e o sub-relato em todos os estudos é a maior limitação de recordatórios ou registros para avaliação do consumo alimentar.

Quanto à média referida de ingestão de energia nos recordatórios, os valores são maiores do que os obtidos no estudo de validação do instrumento usado em 2008–2009, cujos valores foram de 2.017 kcal para homens, com DP = 548, e 1.611 kcal para mulheres, com DP = 452^6^. Assim, pode-se concluir que o sub-relato em 2017–2018 foi provavelmente menor, dado que esses valores para homens e mulheres foram de 2.339 kcal e 1.863 kcal, respectivamente, utilizando as mesmas faixas etárias nos dois estudos.

A análise pormenorizada do consumo de gorduras e açúcares, em relação às mudanças dos métodos e estratégias de análises desses componentes nos dois inquéritos, indicou variação bem pequena para gorduras, mas variação importante para açúcar de adição.

A estratégia de considerar adição de óleo às carnes cozidas, que provavelmente aumentaria o percentual de gordura e, portanto, de energia, não teve impacto nas médias. Possíveis explicações para o achado são as diferenças nos fatores de rendimento, como mudança do peso do alimento após a cocção, e nos padrões de adição de óleo, utilizados para calcular a composição de receita. Esses fatores podem explicar, em parte, variações na densidade energética das carnes refogadas entre a tabela de 2008–2009, baseada principalmente no *software* NDSR, e a TBCA^16^. Além disso, carnes cozidas e refogadas da TBCA diferem em pequena quantidade de óleo de adição, aproximadamente 1 g de óleo de soja para 100 g de alimento, o que também pode ter contribuído para os resultados observados^12^.

Quanto ao açúcar de adição, vale destacar que a intenção de utilizar uma pergunta genérica para inferir a quantidade de açúcar utilizada para adoçar as bebidas tentava recuperar a informação da quantidade de açúcar utilizada pelos indivíduos, uma vez que não havia pergunta sobre o uso de açúcar em alimentos e bebidas específicos. Os resultados encontrados têm relevância no que diz respeito à interpretação dos resultados dos dois inquéritos. Quando se comparam as estimativas do INA 2008–2009 – que não utilizava estratégia de análise para adição de açúcar aos sucos – com o INA 2017–2018, observa-se aumento no consumo de açúcar entre os inquéritos. De forma contrária, quando se comparam as estimativas do INA 2008–2009 considerando a pergunta sobre a frequência de uso de açúcar, verifica-se redução do consumo de açúcar na década analisada. Essas interpretações totalmente distintas dos resultados mostram o quanto a escolha por diferentes estratégias de análise de consumo alimentar pode induzir conclusões conflitantes.

Na publicação do IBGE^17^, foram comparadas as médias de ingestão de açúcar sem considerar a pergunta genérica sobre ingestão de açúcar nas bebidas e em 2017–2018. O açúcar foi incluído também como item de adição, e esse valor entrou no cálculo da média. Comparando os resultados encontrados na publicação do INA 2017–2018, observou-se que a frequência do uso de açúcar para adoçar alimentos e bebidas da população brasileira caiu de 90,8% em 2008–2009 para 85,4% em 2017–2018. Em contrapartida, a preferência por não usar açúcar nem adoçante aumentou de 1,6% para 6,1%, assim como o uso de adoçantes passou de 7,6% para 8,5% no mesmo período^2,3^. Houve também redução no consumo de refrigerantes e refrescos (bebidas que contribuem de forma importante no consumo de açúcar de adição) em ambos os sexos e em todas as faixas etárias e de renda entre os dois inquéritos^3^. Os dados de disponibilidade seguem também esse padrão de redução^17^.

Esses resultados são coerentes com a redução do consumo de açúcar no período investigado, ou seja, reiteram que a comparação mais correta entre os inquéritos consideraria os dados do INA 2008–2009 com a adição de açúcar nas bebidas, utilizando a frequência do uso de açúcar. Além disso, acredita-se que as estimativas do consumo de açúcar considerando-o como item de adição em itens específicos sejam mais próximas da utilização real em comparação com a pergunta genérica. Assim, sugere-se comparar a evolução do consumo de açúcar de adição entre os dois inquéritos utilizando os dados do INA 2008–2009, considerando a pergunta genérica sobre a forma mais frequente de adoçar alimentos, e os dados do INA 2017–2018, utilizando as informações do açúcar como item de adição.

Em geral, as mudanças metodológicas incluídas no INA permitiram aprimorar estimativas de consumo de grupos de alimentos e nutrientes. Além disso, tais mudanças acrescentaram informações mais detalhadas e específicas aos relatos do consumo alimentar, bem como tipificação de refeições e detalhamento maior do consumo fora de casa, o que pode contribuir para avanços na compreensão e análise da alimentação dos brasileiros.
